# Circulating biomarkers of oxidative stress in chronic obstructive pulmonary disease: a systematic review

**DOI:** 10.1186/s12931-016-0471-z

**Published:** 2016-11-14

**Authors:** Elisabetta Zinellu, Angelo Zinellu, Alessandro Giuseppe Fois, Ciriaco Carru, Pietro Pirina

**Affiliations:** 1 Department of Respiratory Diseases, Azienda Ospedaliero Universitaria di Sassari, Sassari, Italy; 2 Department of Biomedical Sciences, University of Sassari, Sassari, Italy; 3 Quality Control Unit, University Hospital of Sassari (AOU SS); Department of Biomedical Sciences, University of Sassari, Sassari, Italy

**Keywords:** Chronic obstructive pulmonary disease, Biomarkers, Oxidative stress, Peripheral blood

## Abstract

Chronic obstructive pulmonary disease (COPD) is a progressive condition characterized by airflow limitation associated with an abnormal inflammatory response of the lungs to noxious particles and gases, caused primarily by cigarette smoking. Increased oxidative burden plays an important role in the pathogenesis of COPD. There is a delicate balance between the toxicity of oxidants and the protective function of the intracellular and extracellular antioxidant defense systems, which is critically important for the maintenance of normal pulmonary functions. Several biomarkers of oxidative stress are available and have been evaluated in COPD. In this review, we summarize the main literature findings about circulating oxidative stress biomarkers, grouped according to their method of detection, measured in COPD subjects.

## Background

COPD is a major and increasing global health problem and is currently the third leading cause of death in the world [1].

**Fig. 1:**
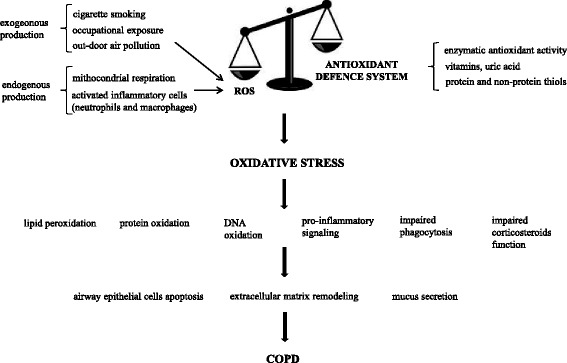
A mechanism showing the central role of oxidative stress in the pathophisiology of COPD. The imbalance between oxidants and antioxidants in favor of oxidants leads to harmful damage. Oxidative stress amplifies the inflammatory response influencing intracellular signaling pathways that drive the release of inflammatory mediators, impairing fagocytosis of apoptotic cells and weakening the ability of corticosteroids to repress proinflammatory genes expression. Inflammation, lipid peroxidation, protein oxidation and DNA damage results in tissue damage, alteration of protein functions and gene expression, remodeling of extracellular matrix and mucus secretion

COPD is defined as a preventable and treatable disease characterized by persistent airflow limitation that is not fully reversible [2]. The airflow limitation is usually progressive and associated with an enhanced chronic inflammatory response of the airways and the lungs to noxious particles or gases. Exacerbations and comorbidities contribute to the overall severity in individual patients [2, 3]. COPD results from the interplay between genetic susceptibility and exposure to environmental stimuli [4]. A well established genetic cause of COPD is α_1_ antitrypsin deficiency [5] whereas, among environmental stimuli, cigarette smoking is the main cause. Other exposures, such as outdoor air pollution, occupational exposure to dusts and fumes, exposure to second-hand smoke, and biomass smoke inhalation might increase the risk of and lead to disease in nonsmokers [6, 7]. Cigarette smoke in particular contains 10^17^ oxidant molecules per puff [8]. Such exposure causes direct injury of airway epithelial cells leading to airway inflammation in which a variety of cells such as neutrophils, macrophages and lymphocytes, are involved. Proteolytic enzymes and reactive oxygen species (ROS) are released and, if not sufficiently counterbalanced by antiproteases and antioxidant factors, will produce further damage [9]. The term ROS indicates a large variety of free oxygen radicals such as superoxide anion (O_2_^−^) and hydroxyl radical (OH^−^), but also derivatives of oxygen that do not contain unpaired electrons, such as hydrogen peroxide (H_2_O_2_). Formation of ROS takes place constantly in every cell during normal metabolic processes. Moreover, activated phagocytic cells such as neutrophils and macrophages produce large amounts of ROS when are stimulated by encounter inhaled particles or other mediators of inflammation [10]. When ROS are produced in excess of the antioxidant defense mechanisms, oxidative stress occurs resulting in harmful effects, including damage to lipids, proteins and DNA. Although the pathogenesis of COPD remains incompletely understood, the central role of oxidative stress in this regard is well established (Fig. 1) [11–14].

Several biomarkers of oxidative stress are available, including ROS themselves. Since ROS are generally too reactive and have a half-life too short to allow their direct measurement in tissues or body fluids, it is more suitable to estimate oxidative stress by measuring their oxidation target products, including lipid peroxidation end products and oxidized proteins, as well as various antioxidants [15]. Regarding COPD, various biomarkers of oxidative stress have been evaluated, both oxidant and antioxidant markers. In this review we summarize the main findings about oxidative stress biomarkers grouped according to their method of detection, evaluated in the blood of COPD patients compared to healthy controls as well as in different stages of the disease. The majority of these studies have considered from mild to very severe COPD while very few studies, to our knowledge, have considered only mild COPD subjects for their analysis.

### Lipid peroxidation products

Lipid peroxidation is the major consequence of oxidative stress and cause of oxidative damage [16].

Many evidences show the association between the levels of these biomarkers and the development of various diseases [15, 17, 18]. Accordingly, lipid peroxidation products have received much attention as biomarkers of oxidative stress.

Lipids are vulnerable to oxidation by both enzymes and nonenzymatic oxidants. The mechanisms and products of lipid peroxidation have been studied extensively [19, 20]. Polyunsaturated fatty acids are very reactive toward oxygen radicals and readily oxidized to produce lipid hydroperoxides and various aldehydes as major products. Cholesterol is also an important substrate of oxidation and its oxidation products are also studied as biomarkers of oxidative stress [21].

Various biomarkers of lipid peroxidation have been developed and applied to biological samples [22]. Among these, malondialdehyde (MDA) and thiobarbituric acid reactive substances (TBARS) remain among the most commonly applied indices of oxidative damage [23, 24]. Several studies have investigated MDA as potential biomarker to assess oxidative stress status in COPD patients using the method of TBARS that involves the reaction of MDA with thiobarbituric acid (TBA) under strong acidic condition and heating, leading to the formation of an adduct which can be easily assessed with a spectrophotometer. One of the most consistent finding across many of these studies was a significant increase in TBARS MDA in COPD patients compared to healthy controls [25–45] (Table 1). Moreover, using this kind of approach some authors have investigated the levels of plasma MDA in parallel with the progression of the disease, observing both an increase with increasing severity of the disease [26, 29, 46, 47] and no differences [40] (Table 1).

**Table 1:** Summary of the most abundant findings about oxidative stress biomarkers in COPD

Oxidative stress biomarker	Method of detection	A	B
MDA	spectrophotometer, following reaction with TBA	↑^25–45^/n.d.^48–53,55^	↑^26,29,46,47^/n.d.^40^
	HPLC, following reaction with TBA	↑^56,57^	
	HPLC	↑^58^	
	spectrophotometer, following reaction with 1-methyl-2-phenylindole	↑^59–61^	↑^59,60^
Lipid peroxides	spectrophotometer, in a solution containing cholesterol-iodide	↑^59^	
	spectrophotometer, following reaction with peroxidase	↑^62^	
Coniugated dienes	spectrophotometer	↑^27,59^	
Oxidized LDL	specific enzyme immunoassay	↑^62^	
8-isoprostane	specific enzyme immunoassay	↑^63^	
Protein carbonyls	spectrophotometer, following the reaction with DNPH	↑^29,35,36,40,59,60,69^/n.d.^73^	↑^59^/n.d^29,36,40^
	immunochemically, following the reaction with DNPH	↑^46,70,71^/n.d. ^72^	
	labeling with tritiated borohydride	↑^52^	↑^53^
AOPP	spectrophotometer	↑^32,40^/n.d^49^	n.d ^40^
Protein SH groups	following reaction with Ellman’s reagent	↓^29,30,36,55,92^/n.d.^39^	n.d ^29,36^
	measuring albumin on a chemistry automated analyzer	↓^56^	
	subtracting GSH from total thiols	↓^53^/n.d^40^	
Plasma reduced GSH	following reaction with Ellman’s reagent	↓^28,34,40,41,43,45,53^/n.d ^55,72^	n.d ^40^
Plasma total glutathione	following reaction with Ellman’s reagent and glutathione reductase		n.d ^47^
Plasma total/oxidized glutathione ratio	following reaction with Ellman’s reagent with glutathione reductase and 1-methyl-2-vinylpyridine	n.d ^95^	
Erythrocyte reduced GSH	following reaction with Ellman’s reagent	↓^26,37^	↓^26^
	following reaction with a reagent different from Ellman’s	n.d.^48^	
Whole blood total glutathione	following reaction with Ellman’s reagent with glutathione reductase and 2-vinylpyridine	↑^29,36^	↑^29^
Plasma total thiols	following reaction with Ellman’s reagent	↓^51^/↑^69^	↓^40^
Erythrocytic SOD activity	Mc Cord and Fridovich assay	↑^36,69^/↓^98,99^/n.d.^33^	n.d ^46^
	Misra and Fridovich assay	↓^27^/↑^27^	
	Marklund and Marklund assay	↓^29^/n.d ^40^	
	Das K. assay	↓^30^	
Plasma SOD activity	Mc Cord and Fridovich assay	↑^61^/↓^32,41^/n.d ^70,71^	n.d ^47^
	ELISA	↓^105^	
Erythrocytic CAT activity	monitoring the decomposition rate of H_2_O_2_	↓^26,29,98,99^/n.d^27,33,36,40^	↓^26^/n.d^40,46^
Plasma CAT activity	monitoring the decomposition rate of H_2_O_2_	↓^34,43^/n.d^47,71^	
	ELISA	n.d^105^	
Erythrocytic GSHPx activity	evaluating the oxidation of NADPH	↓^26,27,29,33,36,98^	↓^46,106^
Plasma GSHPx activity	evaluating the oxidation of NADPH	↓^34,41,43^/↑^36,105^/n.d.^61^	
	assaying the content of reduced glutathione	↑^40^	
Whole blood GSHPx activity	evaluating the oxidation of NADPH	↓^69^	

Compared to healthy controls (column A) and in different stages of COPD (column B). ↑: increased levels; ↓: reduced levels; n.d.: no significant difference

Conversely, few other authors did not find a significant difference in plasma TBARS MDA of COPD patients compared to healthy controls [48–53] (Table 1), while others described a significant increase during acute exacerbations and a return to values comparable to those of controls by the time of discharge after treatment [54]. We investigated MDA levels considering only mild COPD, finding no differences between patients and controls [55] (Table 1). Some authors have analyzed MDA measuring TBA-MDA adduct with high performance liquid chromatography (HPLC) rather than spectrophotometer and finding a significant increase of this biomarker in COPD compared to healthy controls [56, 57] (Table 1). Moreover, measuring free MDA, not as TBA-MDA adduct, by means of HPLC using an ultraviolet spectrophotometric detector at the wavelength of 254 nm, a significant increase of this biomarker has also been found in exacerbated COPD, as well as after treatment of the exacerbation compared to healthy controls [58] (Table 1).

By using a different assay that involves 1-methyl-2-phenylindole, that under acidic and mild-temperature conditions reacts with MDA to yield a stable chromophore with maximal absorbance at 586 nm, an increase of plasma MDA levels has been described [59–61], in some cases also in relation to disease progression [59, 60] (Table 1).

To a lesser extent than MDA, other biomarkers of lipid peroxidation have been studied in plasma of COPD patients of which have been described increased levels compared to controls (Table 1): lipid peroxides, determined spectrophotometrically either using their ability to convert iodide to iodine in a solution containing cholesterol-iodide [59] or following their reaction with peroxidase and a subsequent color production [62]; conjugated dienes, that are formed in the process of lipid peroxidation as a result of a reconfiguration of double bonds and yield a characteristic absorbance peak [27, 59]; oxidized LDL, determined spectrophotometrically with a competitive enzyme-linked immunosorbent assay (ELISA) kit [62] and 8-isoprostane, assayed with a specific enzyme immunoassay kit [63].

### Protein oxidation products

The most abundant byproduct of oxidative damage of proteins is protein carbonylation [64]. The presence of carbonyl groups in proteins is therefore the most commonly used marker of ROS mediated protein oxidation [64, 65], and accumulation of protein carbonyls has been observed in several human diseases [64, 66, 67].

Specifically, carbonyl derivatives (aldehydes and ketones) are formed by reaction of oxidants with lysine, arginine, proline, and threonine residues of the protein side-chains. Moreover, direct reactions of proteins with ROS may also lead to the formation of peptide fragments containing highly reactive carbonyl groups. Proteins containing reactive carbonyl groups can also be generated by secondary reactions of lysine residues of proteins with reducing sugars or their oxidation products and also by reactions of lysine, cysteine, or histidine residues with unsaturated aldehydes formed during the peroxidation of poly-unsaturated fatty acids [68].

The most common and reliable method for determination of carbonyl content is based on the reaction of carbonyl groups with 2,4-dinitrophenylhydrazine (DNPH), which leads to the formation of a stable dinitrophenylhydrazone (DNP) product that can then be detected and quantified spectrophotometrically at 370 nm or immunochemically using specific antibodies to anti-DNP [67].

The approach based on the reaction of carbonyl groups with DNPH has been used in several studies to investigate protein carbonylation levels in the plasma of COPD patients, both detecting DNP product spectrophotometrically [29, 35, 36, 40, 59, 60, 69] and immunochemically [46, 70, 71] (Table 1). All of these studies have described a significant increase of protein carbonyl groups in plasma of COPD patients, that sometimes [59], but not always [29, 36, 40] proceeds in parallel with the progression of the disease (Table 1). On the other hand, few other studies observed no differences in this biomarker levels using the same kind of approach [72, 73] (Table 1).

However, a significant increase in plasma protein carbonylation levels has been described also using another kind of assay, that is by labeling protein carbonyl groups with tritiated borohydride [52] (Table 1). In this study the authors described an increase in the amount of plasma carbonyls in parallel with the progression of COPD. To estimate the degree of oxidant-mediated protein damage in plasma of COPD patients, the presence of advanced oxidation protein products (AOPP) has also been investigated. AOPPs are a family of oxidized, dityrosine-containing, cross linked protein compounds formed by the reaction of plasma proteins, especially albumin, with chlorinated oxidants [74]. Measuring this parameter in a spectrophotometer on a microplate reader [75], both an increase [32, 40] and no difference [49] has been described in plasma of COPD patients compared to controls (Table 1). This marker has been considered in the course of disease progression too, finding no difference throughout the stages of the disease [40] (Table 1).

### Reactive oxygen species (ROS)

Some authors have investigated the production of ROS, in particular O_2_^−^, to explore the levels of oxidative stress in the plasma of COPD patients. A method frequently used to detect O_2_^−^ is based on chemiluminescence. On exposure to O_2_^−^, chemiluminescent probes release a photon, which in turn can be detected by a scintillation counter or a luminometer [76]. Using lucigenin as chemiluminescent probe, an increase in O_2_^−^ production has been described in COPD patients compared to healthy controls [49, 50, 70]. An increase in O_2_^−^ production has also been described using enzymatic assays based on measuring the SOD-inhibitable reduction of cytochrome c determined in a spectrophotometer by the increase in absorbance at 550 nm [36, 39] or using nitroblue tetrazolium that undergoes reduction by O_2_^−^ to form diformazan, a dark insoluble precipitate [32].

### Total oxidative status

Instead of measuring different oxidant species separately, some authors have studied total oxidative status (TOS) in plasma of COPD patients as a marker of oxidative stress, named also total peroxide (TP) or reactive oxygen metabolites (ROMs) [77].

TOS can be evaluated by means of an assay based on the oxidation of ferrous ion to ferric ion by the oxidants present in the sample. The ferric ion makes a colored complex with xylenol orange and the color intensity can be measured spectrophotometrically. Using this assay, an increase in TOS has been described in COPD [32, 78–80]. Other authors have measured ROMs in plasma of COPD patients to test the oxidant ability of the plasma sample towards a particular substance used as an indicator. By means of the so called diacron reactive oxygen metabolites (D-ROM) test, an increase of overall ROMs has been described in COPD patients compared to controls [81, 82].

### Oxidatively damaged DNA

To a lesser extent than other oxidative stress biomarkers, oxidatively damaged DNA has been studied in COPD patients as well. A sensitive method for analyzing oxidative DNA damage is the single-cell gel electrophoresis also known as comet assay, which detects strand breaks [83]. Breaks in DNA allow supercoiled loops of DNA to relax and move out to form what looks like a comet with a tail under the conditions of the assay. The proportion of DNA in the tail is indicative of the frequency of breaks. By means of this assay, a significant increase in DNA damage has been detected in COPD compared to controls [35, 40, 84].

Another commonly used marker for assessing oxidative DNA damage is 8-oxo-7,8-dihydro-2′-deoxyguanosine (8-oxodG). No significant difference in levels of DNA damage, as measured by 8-oxodG by means of ELISA [71] or HPLC [57], has been found.

### Protein and non-protein thiols

Thiols, are a class of organic compounds that contain a sulfhydryl group (−SH). The plasma thiol pool is mainly formed by protein thiols and slightly formed by low molecular-weight thiols, such as cysteine, cysteinylglycine, glutathione (GSH), homocysteine, and γ-glutamylcysteine [85] and are considered a key factor in redox sensitive reactions of plasma [86].

In fact, thiols can undergo oxidation processes in the presence of oxidants to yield a wide range of products, some of which, like disulfides, can revert to thiols with suitable reductants, while others such as sulfinic and sulfonic acids constitute typically final products [85, 87]. Thus, as well as intracellular thiols, such as GSH, are essential in maintaining the highly reduced environment inside the cell, extracellular thiols also constitute an important component of the antioxidant defense system [88, 89].

The most abundant reduced -SH group in plasma is that of human serum albumin, given its high concentrations. The single free cysteinyl thiol of albumin Cys^34^, accounts for about 80 % of reduced thiols in human plasma and is an important scavenger of reactive oxygen and nitrogen species in the vascular compartment [90].

The thiols level is most commonly measured using the classical Ellman’s reagent, 5,5′-dithiobis-(2-nitrobenzoic) acid. This compound is reduced by free thiols in an exchange reaction, forming a mixed disulphide and releasing one molecule of 5-thionitrobenzoic acid, which can be measured at 412 nm [91]. This method of detection has been used in several studies to measure the content of both protein and non-protein SH groups in the peripheral blood of COPD patients.

- ○ *Protein SH groups*: in this regard a significant decrease of this biomarker has been described in COPD compared to healthy controls [29, 30, 36, 92] and even in mild COPD [55] (Table 1). Some authors have investigated this biomarker in the different stages of severity of COPD finding no differences [29, 36]. Interestingly, other authors found a significant reduction of protein SH groups only in exacerbated COPD [39, 54] with a complete restoration by the time of discharge after treatment of exacerbation [54].
   Measuring SH groups by analysis of albumin on a chemistry automated analyzer, a significant reduction of protein thiols has been detected [56] (Table 1). Moreover, measuring protein SH groups by subtracting the GSH content from total thiols measured with Ellman’s reagent, both a significant decrease [53] and no difference between COPD and controls [40] has been detected (Table 1).
- ○ *Non-protein SH groups*: this biomarker has been investigated in plasma, in whole blood and in erythrocytes. A significant decrease in non-protein SH groups, mainly in the form of reduced GSH, has been found in many of the examined studies [28, 34, 40, 41, 43, 45, 53] (Table 1). No differences have been found considering only mild COPD [55] (Table 1). When investigated in the different stages of COPD severity, no difference has been found, either [40] (Table 1). Investigating total GSH (oxidized and reduced) by an assay that employs Ellman’s reagent and glutathione reductase to reduce oxidize glutathione (GSSG) [93], no difference has been found in the different stages of disease severity [47] (Table 1).
   Regarding the levels of erythrocyte reduced GSH, a significant decrease has been described [26, 37] in parallel with the progression of the disease [26] (Table 1), while other authors found no significant difference in this biomarker using a reagent different from Ellman’s to obtain a chromophoric compound which has a maximal absorbance at 400 nm [48] (Table 1).
  Moreover, a significant increase of total GSH, assayed in whole blood of COPD patients using the Ellman’s reagent with glutathione reductase and 2-vinylpyridine to prevent oxidation of GSH during the sample preparation [94], has been described [29, 36] also in parallel with the progression of the disease [29] (Table 1). The same approach, except for using 1-methyl-2-vinylpyridine instead of 2-vinylpyridine, has been used in plasma of COPD patients to investigate total GSH/GSSG ratio and it was found significantly reduced in exacerbation compared to stable COPD, whereas no difference has been found compared to healthy controls [95] (Table 1).

Analyzing protein and non-protein SH groups as total thiols, a significant reduction of this marker has been found in COPD compared to controls [51], as well as with progression of the disease [40], while other authors found that the concentration of total thiols was enhanced in plasma of COPD patients compared to controls [69] (Table 1).

### Antioxidant nutrients

By means of spectrophotometric or chromatographic methods, the plasmatic levels of some antioxidant nutrients such as vitamin A, C and E and α- and β-carotenes that comprise an important aspect of the antioxidant defense system evolved by humans, have been investigated. A decreased level of vitamin C [25, 28, 62] and E [25, 28, 30], as well as no difference in the levels of vitamin A [27], C [40, 48, 95] and E [27] has been described in COPD compared to healthy controls. No differences have also been found in the different stages of severity of disease [96]. A significant reduction of vitamins A, C and E has been described in exacerbated COPD, with a restoration to values similar to that of controls after exacerbation treatment [58]. A significant decrease of α- and β-carotenes has also been described in plasma of COPD patients compared to healthy controls [84].

The levels of essential trace elements, playing a role in oxidant/antioxidant pathways have also been determined. In particular, the plasma levels of selenium (Se) and zinc (Zn), determined by inductively coupled plasma-mass spectroscopy, have been evaluated, finding decreased levels in COPD compared to controls [62]. Measuring Se, Zn, iron (Fe), copper (Cu), potassium (K), rubidium (Rb) and calcium (Ca) by particle-induced X-ray emission, a reduction of K and Se and an increase of Fe, Ca, Cu, Zn and Rb has been described in plasma, while in the blood cells of the same COPD patients a reduction of K and Rb and an increase of the other elements studied has been described [69]. Measuring Cu and Zn by means of atomic absorption spectrophotometry a significant increase of Cu and no difference for Zn has been found [44].

### Uric acid

Uric acid is a powerful antioxidant that protects lipoproteins from oxidation and acts as a powerful scavenger of individual oxygen radicals and hydroxyl radicals. Significantly decreased levels of uric acid have been found by means of an enzymatic method using a colorimetric assay in plasma of COPD subjects compared to healthy controls [97] whereas a significant decrease was found only in very-severe COPD by HPLC with electrochemical detection [95]. No difference has been found using an automated analyzer [49].

### Total antioxidant capacity

Given the difficult to measure each antioxidant separately, several methods have been developed and used to determine the total antioxidant capacities of various biological samples [98]. Some of these methods have been applied to determine the total antioxidant status in the plasma of COPD patients, in particular the FRAP (ferric reducing ability of plasma) and the TEAC (Trolox Equivalent Antioxidant Capacity) assay.

The FRAP assay is based on the ability of plasma to reduce a ferric tripyridyltriazine (Fe^3+^-TPTZ) complex to the ferrous tripyridyltriazine (Fe^2+^-TPTZ), whose intensive blue color can be monitored at 593 nm.

The TEAC assay is based on the inhibition by antioxidants of the absorbance of the radical cation of 2,2′-azinobis (3-ethylbenzothiazoline 6-sulfonate) (ABTS), which has maximal absorption at 660, 734, and 820 nm. The ABTS radical cation is formed when ABTS is incubated with the peroxidase metmyoglobin and H_2_O_2_. Upon the addition of a plasma sample, the oxidative reactions are suppressed by the antioxidant capacity of the plasma, preventing the color change.

Using the FRAP assay a significant decrease of total antioxidant capacity has been found in COPD patients compared to controls [29, 36] in relation also to disease progression [29, 36, 96].

Using the TEAC assay, a significant decrease of total antioxidant capacity [40, 54, 92, 97, 99, 100] as well as no significant difference [49] has been described in COPD compared to controls. Moreover, other authors found a significant reduction only in exacerbated COPD [39]. The TEAC assay has been used also to investigate this biomarker in relation to disease progression, finding no difference throughout the various stages of the disease [40]. By means of another assay based on preventing the oxidation of ortho-dianisidine molecules into dianisidyl radicals by hydroxyl radicals, a significant decrease of total antioxidant potential has been described in COPD compared to controls [79]. A significant decrease of total antioxidant capacity has also been found by means of another kind of assay [31, 32] that is based on preventing the oxidation of ortho-dianisidine molecules into dianisidyl radicals by hydroxyl radicals OH^−^.

### Enzymatic antioxidant activity

Some antioxidant enzymes have been widely studied in blood of COPD patients such as superoxide dismutase (SOD), catalase and glutathione peroxidase (GSH-Px). To a lesser extent, the activities of glutathione-S-transferase (GST), paraoxonase 1 (PON1) and ceruloplasmin ferroxidase have also been studied.

### SOD activity

SOD catalyzes dismutation of the O_2_^−^ to molecular oxygen and H_2_O_2_. In most studies SOD activity has been measured in COPD erythrocytes with the Mc Cord and Fridovich assay [101] finding different results (Table 1). On this method xanthine and xanthine oxidase are used to generate O_2_^−^ and 2-(4- iodophenyl)-3-(4-nitrophenol)-5-phenyltetrazolium chloride which reacts with O_2_^−^ to form red formazan dye. SOD inhibits this reaction and the activity is measured as percent inhibition. Using this method, either an increase [36, 69] or a decrease [99, 100] of erythrocytes SOD activity, as well as no difference [33] has been found in COPD versus controls (Table 1). No differences have also been found comparing moderate and severe COPD [46] (Table 1). Moreover, an increase in erythrocytes SOD activity has been observed only in exacerbated COPD [38].

Other assays measure SOD activity taking advantage of its ability to inhibit various reactions such as the auto oxidation of epinephrine to adrenochrome [102], the auto oxidation of pyrogallol [103] and the nitrite formation subsequent to reactions of O_2_- with hydroxylamine hydrochloride [104]. The use of these assays brought different results. It has been found both a decrease [26, 29, 30] and an increase [27] of erythrocyte SOD activity as well as no difference compared to controls [40] (Table 1).

SOD activity has been determined also in plasma of COPD patients using assays based on the inhibition of red formazan dye formation, finding an increase of SOD activity [61], a decrease of SOD activity [32, 41] and no difference [70, 71] compared to controls, as well as no difference throughout the stages of the disease [47] (Table 1). Estimating SOD with an ELISA kit, a significant reduction has been found in COPD subjects compared to controls [105] (Table 1).

### CATALASE activity

Catalase is involved in the detoxification of H_2_O_2_ into molecular oxygen and water. Its activity has been measured in COPD erythrocytes using different methods based on monitoring the decomposition rate of H_2_O_2_ at 240 nm. In such way, both a decrease [26, 29, 99, 100] and no difference [27, 33, 36, 40] of catalase activity has been observed in COPD compared to controls (Table 1). Studying catalase activity in relation to disease progression has also brought different results, namely a significant decrease from moderate to severe COPD [26] as well as no difference either comparing moderate and severe COPD [46] or comparing all the stages of the disease [40] (Table 1). Catalase activity has been measured also in plasma in few studies where both a decrease [34, 43] and no significant difference [47, 71] has been observed in COPD compared to controls (Table 1). Moreover, estimating the enzyme activity with an ELISA kit, no significant difference has been found [105] (Table 1).

### GSHPx activity

GSHPx activity converts reduced GSH to GSSG while reducing organic peroxides into their corresponding alcohols or H_2_O_2_ into water. Its activity has been measured in plasma, in total blood and especially in erythrocytes evaluating at 340 nm the oxidation of nicotinamide adenine dinucleotide phosphate (NADPH), a coenzyme in the reaction catalyzed by glutathione reductase that reduces GSSG formed during the activities catalized by GSHPx.

In most of the studies a decreased GSHPx activity in COPD erythrocytes has been described compared to controls [26, 27, 29, 33, 36, 99] as well as in relation to disease severity [46, 106] (Table 1).

A decreased GSHPx activity has been observed analyzing COPD total blood [69] and plasma [34, 41, 43] (Table 1). In plasma, no difference in GSHPx activity [61] as well as an increase either monitoring the rate of NADPH oxidation [36, 105] or assaying the content of reduced GSH [40] has also been observed (Table 1).

### GST, PON 1 and ceruloplasmin ferroxidase activities

GST catalyzes the inactivation of reactive electrophiles through their conjugation with GSH while PON1, an esterase associated with high-density lipoprotein (HDL), protects against the toxicity of some organophosphates and contributes to the antioxidant protection conferred by HDL on low-density lipoprotein oxidation. GST activity has been studied using 1-chloro- 2,4-dinitrobenzene as an artificial substrate in plasma of COPD finding a decreased activity [99] and no differences compared to controls [61] as well as in erythrocytes [26] where a decreased enzymatic activity has been described.

PON1 activity has been evaluated in plasma of COPD using a two-substrate activity (paraoxon/diazoxon) method or by the hydrolysis of paraoxon alone. Using the first method no significant difference has been described in COPD compared to controls [32, 78]. Using the paraoxon method, a significant decreased enzyme activity has been described, either in COPD versus controls [44] and in the different stages of severity of disease [59]. No significant difference was found when only mild COPD was considered [55].

Ceruloplasmin is an important contributor to plasma antioxidant activity that includes ferroxidase activity, GSH-Px activity and the ability to scavenge ROS [107]. Its oxidase activity has been investigated in plasma of COPD subjects by means of an assay that works on its ability to oxidize ferrous ion to ferric ion, complexing with a chromogen that can be measured at 376 nm. With this technique, a significant increase of the enzyme activity in COPD has been found [105] as well as no significant difference [100]. By means of immunonephelometry on an automated analyzer a significant increase of ceruloplasmin was found in COPD [56].

### γ‑glutamyltransferase (GGT) activity

GGT is a plasma membrane enzyme, which is involved in antioxidant glutathione resynthesis. Serum GGT levels are increased in a number of diseases that are known to have oxidative stress in the pathogenesis, suggesting that GGT levels can be considered a marker of oxidative stress [108, 109]. GGT activity has been investigated in plasma of COPD patients. By using standardized methods on automatic analyzer, a significant increase of the enzyme activity has been found in COPD compared to healthy controls [110] as well as no difference [111]. A significant increase of GGT activity has also been found in exacerbated COPD compared to stable state [112] whereas no differences have been found in the different stages of the severity of the disease [110].

## Conclusions

In this review we have summarized the main findings about the most studied circulating biomarkers of oxidative stress in COPD subjects, grouping them depending on the method of detection that could be useful for those who wants to deal with this issue.

Although oxidative stress has been largely studied in COPD, we still lack standardized biomarkers useful in diagnosis and in monitoring the progression of the disease. What emerges from literature is that lipid peroxidation products are the most studied as biomarkers of oxidative stress in COPD, especially MDA. In most cases it has been reported an increase of this marker either as MDA or as lipid peroxides, 8-isoprostane, conjugated dienes or oxidized LDL.

Other oxidative stress biomarkers that have often been studied in plasma of COPD patients are protein oxidation products, in particular protein carbonyls that have been described increased in about 85 % of the studies. A marker of oxidative stress that, to our knowledge, has been described always increased in the examined case–control studies is superoxide anion, even if different assays have been used to investigate this marker. In addition, total oxidative status has been described always increased as well, by means of two types of assays and also in mild COPD.

Interestingly, concordant results have been found for these oxidative stress biomarkers at least in 80 %, if not in all of the examined studies, even using different methods of analysis. The explanation of why, in some cases, the remaining 20 % of the studies gave different results is probably due to a different preanalytical approach, e.g. the sample collection, the time that elapses between collection of the sample and separation of serum from the blood cells, and the storage of the sample (temperature storage, container used for storage and repeated thawing). We must also consider the biological variability, due to age, sex, race and genetic selection. All of these variables unavoidably influence the measurement. Regarding antioxidant markers, protein and non protein SH groups have been largely investigated giving different results especially for the latters that have been studied in plasma, in erythrocytes and in whole blood. Nevertheless, protein SH groups and plasma reduced GSH have been described reduced in about the 80 % of the examined studies. Considering total antioxidant capacity, 85 % of the examined studies have reported a significant decrease of this parameter in COPD using different methods of analysis. On the contrary, conflicting results have been found analyzing the levels of antioxidant nutrients, such as vitamins A, C and E, and the enzymatic antioxidant activities. In fact, some authors have found a significant decrease of these biomarkers, while others have found no significant difference.

These conflicting findings could be due not only to the reasons explained above regarding the pre-analytical steps, but also to the fact that studies were carried out in different populations, and that there may be differences and inter-individual variations in antioxidant capacity, also due to cigarette smoke and its effect on the imbalance between oxidants and antioxidants.

From an overall point of view, despite the difficulties in reproducing the same results using different assays in different research laboratories, the findings summarized in this review highlight that literature is quite concordant in concluding that the blood of COPD patients presents an increase of oxidants and a decrease in some antioxidant defences compared to controls.

Up to now a large number of biomarkers have been evaluated in COPD, but their relative importance is not yet clearly understood. Hence, there is clearly the need to identify suitable biomarkers able to detect disease, to monitor disease progression, exacerbations and response to therapy. For this purpose, the choice of peripheral blood among other biological matrices, seems to be more appropriate given the non invasiveness of the blood sampling, its property of easily allowing repeated measurements and its effectiveness in monitoring systemic effects such as oxidative stress. Surely, further research is needed to validate such markers and a great effort should be done to better characterize subjects under study and to understand the issues that likely influence the measurements. Anyhow, this review is an important step in this context providing a comprehensive overview of the oxidative stress biomarkers evaluated in the blood of COPD subjects, stressing their potential utility in supporting diagnostic and therapeutic decisions.

## Abbreviations

8-oxodG: 8-oxo-7,8-dihydro-2′-deoxyguanosine
ABTS: 2,2′-azinobis (3-ethylbenzothiazoline 6-sulfonate)
AOPP: Advanced oxidation protein products
Ca: Calcium
CAT: Catalase
COPD: Chronic obstructive pulmonary disease
Cu: Copper
DNP: Dinitrophenylhydrazone
DNPH: 2,4-dinitrophenylhydrazine
ELISA: Enzyme linked immunosorbent assay
Fe: Iron
FRAP: Ferric reducing ability of plasma
GSH: Glutathione
GSH-Px: Glutathione peroxidase
GSSG: Oxidize glutathione
GST: Glutathione-S-transferase
H_2_O_2_: Hydrogen peroxide
HDL: High-density lipoprotein
HPLC: High performance liquid chromatography
K: Potassium
MDA: Malondialdehyde
NADPH: Nicotinamide adenine dinucleotide phosphate
O_2_^−^: Superoxide anion
OH^−^: Hydroxyl radical
PON1: Paraoxonase 1
Rb: Rubidium
ROMs: Reactive oxygen metabolites
ROS: Reactive oxygen species
Se: Selenium
−SH: Sulfhydryl group
SOD: Superoxide dismutase
TBA: Thiobarbituric acid
TBARS: Thiobarbituric acid reactive substances
TEAC: Trolox Equivalent Antioxidant Capacity
TOS: Total oxidative status
TP: Total peroxide
Zn: Zinc
